# Correction: Patano et al. Mandibular Crowding: Diagnosis and Management—A Scoping Review. *J. Pers. Med.* 2023, *13*, 774

**DOI:** 10.3390/jpm15100446

**Published:** 2025-09-23

**Authors:** Assunta Patano, Giuseppina Malcangi, Alessio Danilo Inchingolo, Grazia Garofoli, Nicole De Leonardis, Daniela Azzollini, Giulia Latini, Antonio Mancini, Vincenzo Carpentiere, Claudia Laudadio, Francesco Inchingolo, Silvia D’Agostino, Daniela Di Venere, Gianluca Martino Tartaglia, Marco Dolci, Gianna Dipalma, Angelo Michele Inchingolo

**Affiliations:** 1 Department of Interdisciplinary Medicine, University of Bari “Aldo Moro”, 70124 Bari, Italy; assuntapatano@gmail.com (A.P.); giuseppinamalcangi@libero.it (G.M.); ad.inchingolo@libero.it (A.D.I.); graziagarofoli.g@libero.it (G.G.); nicoledeleonardis@outlook.it (N.D.L.); daniela.azzollini93@gmail.com (D.A.); dr.giulialatini@gmail.com (G.L.); dr.antonio.mancini@gmail.com (A.M.); vincenzo.carpentiere@gmail.com (V.C.); c.lauda@hotmail.it (C.L.); silviadagostino00@gmail.com (S.D.); daniela.divenere@uniba.it (D.D.V.); giannadipalma@tiscali.it (G.D.); angeloinchingolo@gmail.com (A.M.I.); 2 Department of Medical, Oral and Biotechnological Sciences, University G. D’Annunzio, 66100 Chieti, Italy; marco.dolci@unich.it; 3 Department of Biomedical, Surgical and Dental Sciences, School of Dentistry, University of Milan, 20100 Milan, Italy; 4 UOC Maxillo-Facial Surgery and Dentistry, Fondazione IRCCS Cà Granda, Ospedale Maggiore Policlinico, 20100 Milan, Italy

Following discussions between the Editorial Board and the authors, the original published references numbered 47 and 48 have been removed from this paper [1], and reference number 47 has been replaced by the following reference:

47. Myser, S.A.; Campbell, P.M.; Boley, J.; Buschang, P.H. Long-term stability: Postretention changes of the mandibular anterior teeth. *Am. J. Orthod. Dentofac. Orthop.* **2013**, *144*, 420–429.

Along with the deletion of reference 48, references 49–52 were re-numbered as references 48–51. And the citations of references 49–52 in the main text have been updated with the numbers 48–51.

Corresponding updates have also been made to the References section and to the citations throughout the manuscript. The authors state that the scientific conclusions are unaffected. This correction was approved by the Academic Editor. The original publication has also been updated.
